# One-step Maskless Fabrication and Optical Characterization of Silicon Surfaces with Antireflective Properties and a White Color Appearance

**DOI:** 10.1038/srep35183

**Published:** 2016-10-11

**Authors:** Ling Schneider, Nikolaj A. Feidenhans’l, Agnieszka Telecka, Rafael J. Taboryski

**Affiliations:** 1 Department of Micro- and Nanotechnology, Technical University of Denmark, 2800 Kongens Lyngby, Denmark; 2 Danish Fundamental Metrology A/S, Matematiktorvet 307, 2800 Kongens Lyngby, Denmark

## Abstract

We report a simple one-step maskless fabrication of inverted pyramids on silicon wafers by reactive ion etching. The fabricated surface structures exhibit excellent anti-reflective properties: The total reflectance of the nano inverted pyramids fabricated by our method can be as low as 12% without any anti-reflective layers, and down to only 0.33% with a silicon nitride coating. The results from angle resolved scattering measurements indicate that the existence of triple reflections is responsible for the reduced reflectance. The surfaces with the nano inverted pyramids also exhibit a distinct milky white color.

Silicon is abundant in nature, and has been widely used in the semiconductor industry. By texturing silicon surfaces, it is possible to obtain samples of dual- or multi-functions that are attractive in many fields. Recently, there has been increasing interest in using “black silicon” fabricated by reactive ion etching (RIE) to enhance the light trapping efficiency of photovoltaic devices^1^,^2^,^3^,^4^,^5^,^6^. The RIE fabricated silicon typically consists of needle-like or cone-shape structures that scatter the incident light, which is the reason for the black or brownish color of the surface^3^,^7^,^8^,^9^,^10^,^11^. Though highly anti-reflective, the black silicon suffers from high surface recombination losses caused by its extremely high surface area, which as a result limits the increase of the external quantum efficiency of photovoltaic devices^12^,^13^. Recently, Savin *et al*. reported that it is possible to chemically and electrically passivate the black silicon to reduce the charge recombination by depositing an alumina layer^14^. Alternatively, it has been reported that inverted pyramids have lower surface areas than upright pyramids, yet still good light trapping properties^12^,^13^,^15^,^16^,^17^,^18^,^19^,^20^. These inverted pyramids are typically fabricated either by colloidal lithography^21^,^22^, or by combining interference lithography and wet silicon etching^13^,^16^,^17^,^19^. Both methods are complex and require masks or templates for the fabrication, which increases the fabrication costs. Wang *et al*. recently reported on using a maskless copper assisted acid etching method to fabricate micro inverted pyramids on a silicon surface^20^. Nonetheless, as copper is a deep donor for silicon, the use of copper could potentially cause contamination to the fabricated samples and other processes performed in the same cleanroom.

In this letter we report a simple one step maskless method to fabricate semi-periodic nano inverted pyramids on silicon wafer surfaces by RIE. RIE is a dry etching technique that can be used to structure silicon surfaces through the combined effect of a corrosive gas (SF_6_ or CH_4_) and a passivating gas (O_2_) without additional masks^7^,^8^. We will show that by fine tuning the etching parameters, we are able to fabricate nano inverted pyramids of different sizes and regularities. The total reflectance of the inverted pyramids fabricated by our method can be as low as 12% without any anti-reflective layers, and down to only 0.33% with silicon nitride (SiN_x_) coatings. The surfaces with the uncoated nano inverted pyramids also exhibit a very interesting milky white color, which to the best of our knowledge has not been reported yet. As reactive ion etching (RIE) is widely used in the semi-conducting industry, our technique will have high potential not only in the photovoltaic but also other industries, where surfaces of special optical properties are desired. Moreover, this technique will lead towards cost-effective large-scale productions of inverted pyramidal structures.

The structure fabricated by RIE is determined by complex reactions happening inside the RIE chamber, which according to many studies depend mainly on the gas ratio, chamber temperature, chamber pressure, and the platen power^2^,^3^,^5^,^10^,^11^,^23^,^24^,^25^,^26^. The absolute gas flow on the other hand does not seem to have much influence on the formed structures. However, in most of the aforementioned studies, the change of the gas flow rate is only within 10%, which perhaps is too small to influence the structure formation. To better understand the parameter influence and better control the etched structures, in this study we fixed the chamber temperature (−19 °C), chamber pressure (38 mTorr), and the platen power (6 W), and varied the flow rates *Q* (in sccm) of SF_6_ and O_2_ and the etching time *t* (in min). The total gas flow rate can be 12 times higher for the batches with the highest rate than for those with the lowest rate. For simplification, we denote our samples of different etching parameters as 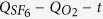.

By changing the SF_6_ and O_2_ flow rates and the etching time, we are able to obtain waferscale samples of very different surface structures with good reproducibility (samples 55-10-10, 50-10-15, and 50-10-20 were reproduced five times), as presented in Fig. 1. The fabricated structures range from flat surfaces with random particles (500-100-7 and 100-20-7), winding micro-trenches (60-10-7), non-periodic holes (40-10-7), to semi-periodic inverted pyramids (50-10-7 and 55-10-10). It is worth mentioning that the gas ratio 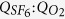 and the etching time *t* of samples 500-100-7 and 50-10-7 are the same, while the resulting structures of the two samples are very different: the surface of sample 500-100-7 is almost flat with only some random particles of irregular shape; while the surface of sample 50-10-7 already has inverted pyramidal holes. Apparently, in our study the influence of the total flow rate on the structure formation can be substantial. The dominant effect seems to be the residence time of the gas molecules in the active etching zone, where the plasma is generated. The average residence time τ of a gas inside the RIE chamber can be defined as: τ = *pV*/*Q*^26^, where *p* is the pressure inside the chamber, *V* the plasma volume, and *Q* the gas flow rate. As we used fixed chamber pressures in all experiments, the throttle valve opened more for higher gas flow rates to maintain the chamber pressure than the lower ones. For a comparison, the average gas residence time of sample 500-100-7 is only 0.02 s (Fig. S2), while τ of sample 50-10-7 is 0.21 s. Hence, for the high gas flow rates, gases pass the active etching zone so fast that there is not enough time for them to dissociate and react with the sample surface in the chamber, leading to an almost flat surface (sample 500-100-7). Similar observations were also reported by Jansen *et al*.^26^.

Figure 2b,c are representative spectra of the specular and total reflectance of all fabricated types. Three samples with inverted pyramidal structures (50-10-15, 50-10-20, and 55-10-10) are among those of the lowest specular and total reflectance (Fig. 2a,b). The total reflectance of sample 55-10-10 (without anti-reflective coating) is lower than 20% at shorter wavelength, and as low as 12% at near IR, which is lower than many reported values of uncoated nano/micro inverted pyramids made by more complex methods^17^,^27^,^28^. The inverted pyramids of this sample (55-10-10) are almost arranged in a hexagonal array, and yet with a predominantly quadratic base having side lengths of around 600 nm and a pitch distance of around 700 nm, much smaller than those reported by Wang *et al*.^20^. The total reflection of these three samples is further reduced down to only 0.33% after coating with the anti-reflective layer (~80 nm, Fig. 2c), which is also used as a passivation layer to reduce the surface recombination rate and to increase the effective carrier lifetime in photovoltaic devices^13^,^29^,^30^.

Figure 2d shows the angle resolved scattering (ARS) spectra of sample 55-10-10 without and with SiN_x_ coating. The solid-dash lines are the respective cosine fittings of the scattered intensity at angle *θ*_*s*_, *ARS* = *A* · cos(*θ*_*s*_). Here A is a prefactor, and *θ*_*s*_ is the angle between the detector and the plane normal to the sample surface (sample normal). The ARS curve of the uncoated 55-10-10 fits well at large angles but deviates from the cosine function at small angles. There are two peaks at *θ*_*s*_ ≈ −46° and *θ*_*s*_ ≈ −21°, which could result from double and triple reflections of incoming beam rays by the facets of the inverted pyramids, as illustrated in Fig. 2e. The average plane inclination of the inverted pyramids is *α* ≈ 56.5°, as measured by ImageJ on SEM images. This angle is close to the well-known angle of 54.7° usually obtained for anisotropic wet etching of <100> Si surfaces with potassium hydroxide (KOH). By simple trigonometry, the angle of the second reflection to the sample normal can be derived *β* ≈ 46°, and the angle of the third reflection to the sample normal γ ≈ 21°. The values of these two angles fit well with the positions of the two peaks observed in Fig. 2d. The intensity difference between the two peaks can have two origins: 1. The reflection fraction of flat silicon surfaces varies at different angles of incidence according to the Fresnel equation; 2. For an inverted pyramid of *D* ≈ 609 nm and *α* ≈ 56.5° as presented in Fig. 2e, only 15% of the incoming beam (per facet) falls in an area, where a third reflection could happen; the rest would be either reflected out of the inverted pyramid or absorbed by the pyramid (refracted). Similar hypothesis of triple reflections of inverted pyramids was also suggested by other groups^17^,^20^. Due to the restriction of our experimental setup, light scattered to angles lower than 17° to the sample normal could not be measured, which makes it difficult numerically to fit the two peaks or calculate their integral precisely. However, the ARS ~ *θ*_*s*_ curves of the coated surfaces fit the cosine function very well (Figs 2d and S4). It is worth mentioning that structures fabricated by RIE are not defect free, which might introduce additional recombination sites when compared to defect free structures^31^. Nonetheless, the decrease in the surface area, and the possibility to passivate the surface by introducing alumina or SiN_x_ still makes the inverted pyramids fabricated by our method promising for photovoltaic applications.

In addition to being anti-reflective, some of our inverted pyramids appear “white” or greyish, which is distinctly different from the classical black or brownish silicon wafers made by RIE^3^,^7^,^25^,^26^. A clear demonstration of the special color of these nano inverted pyramids is presented by a photo of a white “DTU logo” (approx. 3 mm × 4 mm) on a black background (Fig. 3a). The white DTU logo was fabricated using the same parameters as sample 55-10-10 on a black silicon wafer of nano spikes. The detailed fabrication procedure and parameters can be found in the experimental section and the supporting information. The SEM image in the white area clearly shows the inverted pyramids are fabricated, though a bit larger (pitch distance up to 1 μm) than the structures fabricated on the polished silicon wafer (Figs 1 and 3c and S5), which might be caused by the incomplete removal of nanograss on the black silicon surface. The color of the logo clearly appears white to human eyes, as compared to the white A4 paper under the sample. The intensity profiles (extracted by ImageJ) of the RGB colors (Fig. 3d) along the red line on Fig. 3b,e) overlap well with each other, which further verifies the white color of the DTU logo^32^. There are some oscillations (period ∼ 4 μm) on the color intensity profile, which might stem from defects of similar period in the area (Fig. S5). These defects might be caused by the inhomogeneous removal of the pre-etched nano spikes in the logo area by RIE, as no such structural irregularities are observed on samples fabricated directly from polished Si wafers (Fig. 1).

In summary, we have demonstrated a maskless one-step method to fabricate nano inverted pyramids on silicon wafers by RIE. These structures are very sensitive to the parameters used in RIE, and the total gas flow rate can be substantial to the structure formation. The uncoated inverted pyramids have the lowest specular and total reflectance among all fabricated samples. The low total reflectance of these inverted pyramids is probably caused by a third reflection of the incoming beam by the pyramid facet. The total reflectance of the inverted pyramids is further reduced by an order of 40 after coating with a layer of SiN_x_, which makes the inverted pyramid fabricated by RIE very promising for photovoltaic applications. As demonstrated by the white DTU logo, some of the inverted pyramids have a milky white color, which could be interesting in other industries. As RIE is an industrialized standardized silicon dry etching technique, our method will pave the way to large scale, cost-effective mass production of nano inverted pyramids.

## Experimental Section

### Silicon surface texturing

All samples were textured by reactive ion etching (RIE, Pegasus D-RIE, STS, UK) on crystalline 100 mm silicon wafers (n-doped (phosphorous), resistivity 10–100 Ωcm, (100)), at different SF_6_ and O_2_ flow rates, platen power, temperature, and etching time. The white DTU logo with black background was fabricated by RIE on a pre-etched black silicon substrate coated with patterned photoresist of the DTU logo. The detailed fabrication parameters and process flow can be found in the supporting information. After RIE, all samples were cleaned by N_2_/O_2_ plasma (N_2_ 400 sccm, O_2_ 70 sccm, power 1000 W) for 30 min. For the coated samples, a layer of approx. 80 nm silicon nitride (SiN_x_) was immediately deposited on the structured surface by plasma enhanced chemical vapor deposition (PECVD, SPTS, UK) after the plasma cleaning. The film thickness of SiN_x_ was tested by ellipsometry (Ellipsometer VASE, J.A. Woollam Co., Inc, USA) on a parallel plain wafer coated with the same parameters.

### Structure characterization

All samples were characterized by scanning electron microscopy (SEM, Supra 40 VP, Carl Zeiss AG, Germany) at both cross-sectional and surface views. The dimension of the structures was measured by ImageJ (version 1.49s).

### Optical characterization

The specular reflection was measured by multiple angle reflectometry (Film Tek 4000, scientific computing international, Carlsbad, USA) at normal and 70° incidence. The total reflectance was measured by optical spectrometer (OE65000, Ocean Optics spectrometer, USA) with an integrating sphere of 8° incidence (AvaSphere-50, Avantes, UK). The total reflectance measurement was calibrated by a white Lambertian scatter (SRS-99-020, LabSphere, USA). The scattering distribution of the samples was measured with an angular scatterometer, where a photodetector (New Focus Model 2032, Newport, USA) was rotated in a circular arc around the sample and the scattering intensity was evaluated in steps of 0.1°. A 40 mW argon-ion laser of 488 nm (60X, American Laser Corporation, USA) was used as the incident light source. The detailed description of the setup can be found in a previous publication^33^. Canon EOS 5D Mark II camera was used to shoot the photos of the white DTU logo on the black silicon wafer. Except for the auto white balance function, no further post processing was used on the photos. The RGB profile of the photo was extracted by ImageJ.

The silicon surface texturing, SEM characterizations, and the specular reflection measurements were carried out in a class 10–100 cleanroom (Danchip, DTU, Denmark).

## Additional Information

**How to cite this article**: Schneider, L. *et al*. One-step Maskless Fabrication and Optical Characterization of Silicon Surfaces with Antireflective Properties and a White Color Appearance. *Sci. Rep.* **6**, 35183; doi: 10.1038/srep35183 (2016).

## Supplementary Material

Supplementary Information

## Figures and Tables

**Figure 1:**
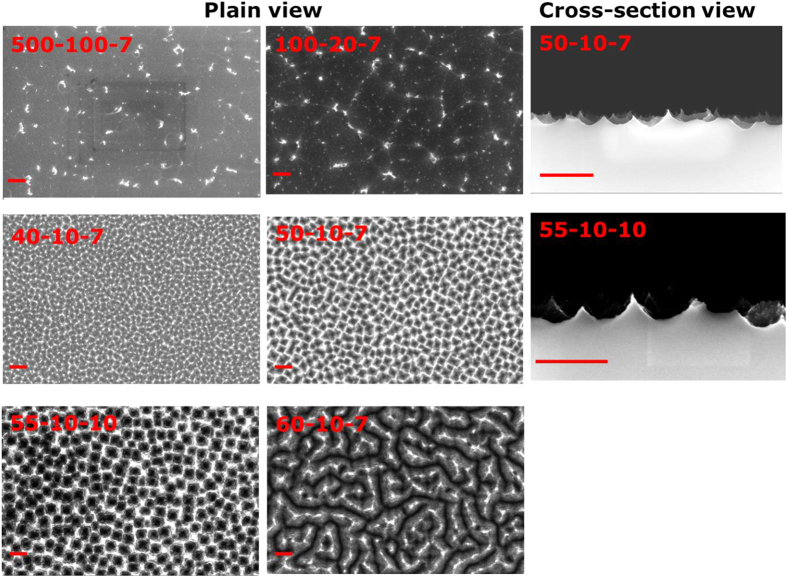
SEM images of structures fabricated at different gas flow rates and etching times. The scale bars are 1 μm.

**Figure 2:**
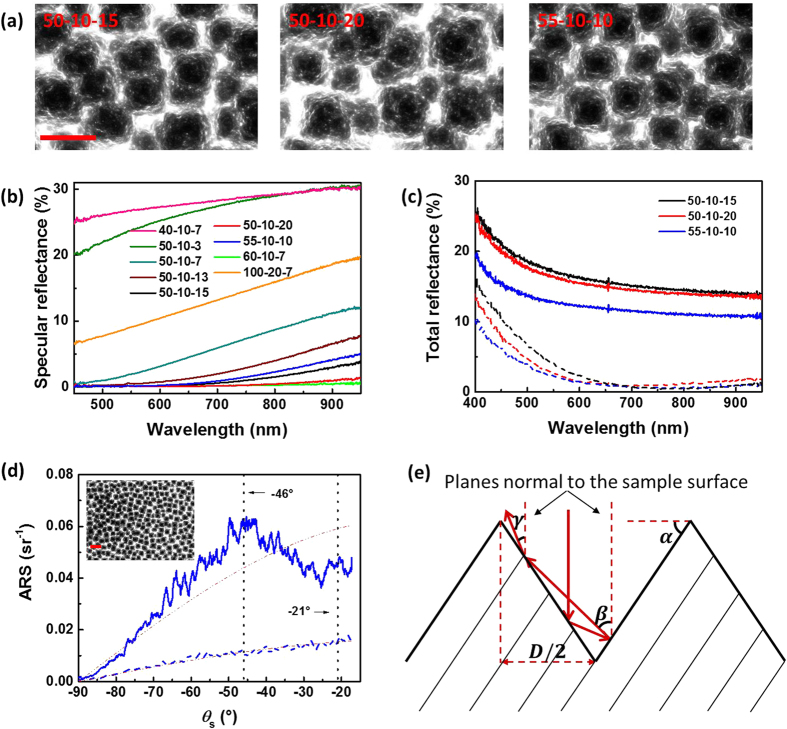
(**a**) SEM images of uncoated inverted pyramids. (**b**) Specular reflectance of uncoated samples measured at normal incidence. (**c**) Total reflectance of inverted pyramids without (solid line) and with (dash line) ~80 nm SiN_x_ coating measured with an integrating sphere. (**d**) Angle resolved scattering (ARS) spectra of sample 55-10-10 without (blue solid line) and with (blue dash line) SiN_x_ coating measured by a diode laser of 488 nm. The red dash-dot lines are cosine fittings of the corresponding ARS curves. Inset: SEM image of uncoated 55-10-10. (**e**) Sketch of possible reflections of the incident light beam by one inverted pyramid. The scale bars are 1 μm.

**Figure 3:**
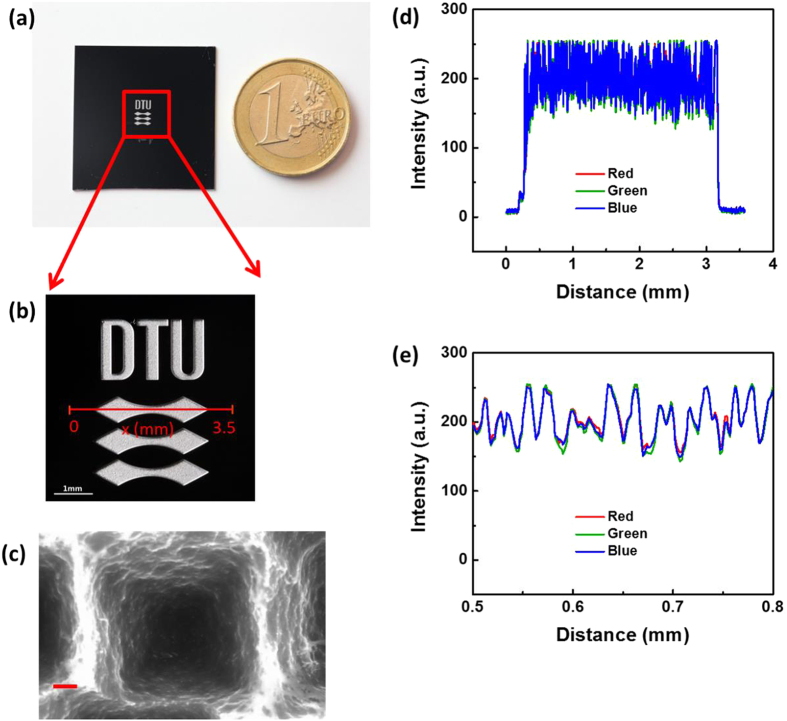
(**a**) Photo of a black silicon wafer with the as fabricated white DTU logo, in reference to a one EUR coin. Both the wafer and the coin were placed on a white A4 paper for color reference. (**b**) The zoomed photo of the logo area. (**c**) SEM image of one single inverted pyramid in the DTU logo area. The scale bar is 200 nm. (**d**) The intensity profile of RGB colors (red, green, and blue) along the red line indicated on (**b**). (**e**) The zoomed intensity profile of RGB colors at x = 0.5–0.8 mm.
