# Supplementary material for: One-step Maskless Fabrication and Optical Characterization of Silicon Surfaces with Antireflective Properties and a White Color Appearance
Source: Sci Rep. 2016 Oct 11;6:35183. doi: 10.1038/srep35183 (PMC5057096; doi:10.1038/srep35183)
Supplement: Supplementary Information [file srep35183-s1.pdf]

Supporting information for:

One-step Maskless Fabrication and Optical Characterization of Silicon Surfaces with Antireflective Properties and a White Color Appearance

*Ling Schneider[1,*], Nikolaj A. Feidenhans'l[2], Agnieszka Telecka[1], Rafael J. Taboryski[1,*]*

[1] Department of Micro- and Nanotechnology, Technical University of Denmark, 2800 Kongens Lyngby, Denmark

[2] Danish Fundamental Metrology A/S, Matematiktorvet 307, 2800 Kongens Lyngby, Denmark

* lisu@nanotech.dtu.dk, rata@nanotech.dtu.dk

**Table S1**. RIE parameters of different samples and the diameter of selective samples. Sample 70-100-40 is black.

<table>
<tr><th>sample</th><th>chamber pressure</th><th>platen power</th><th>$SF_6$</th><th>$O_2$</th><th>temperature</th><th>etching time</th><th>Diameter</th></tr>
<tr><td></td><td>mTorr</td><td>W</td><td>sccm</td><td>sccm</td><td>°C</td><td>min</td><td>nm</td></tr>
<tr><td>100-20-7</td><td rowspan="10">38</td><td rowspan="9">6</td><td>100</td><td>20</td><td rowspan="9">-19</td><td>7</td><td></td></tr>
<tr><td>500-100-7</td><td>500</td><td>100</td><td>7</td><td></td></tr>
<tr><td>60-10-7</td><td>60</td><td>10</td><td>7</td><td></td></tr>
<tr><td>40-10-7</td><td>40</td><td>10</td><td>7</td><td></td></tr>
<tr><td>50-10-3</td><td>50</td><td>10</td><td>3</td><td></td></tr>
<tr><td>50-10-7</td><td>50</td><td>10</td><td>7</td><td>514</td></tr>
<tr><td>50-10-15</td><td>50</td><td>10</td><td>15</td><td>484</td></tr>
<tr><td>50-10-20</td><td>50</td><td>10</td><td>20</td><td>560</td></tr>
<tr><td>55-10-10</td><td>55</td><td>10</td><td>10</td><td>609</td></tr>
<tr><td>70-100-40</td><td>20</td><td>70</td><td>100</td><td>-10</td><td>40</td><td></td></tr>
</table>

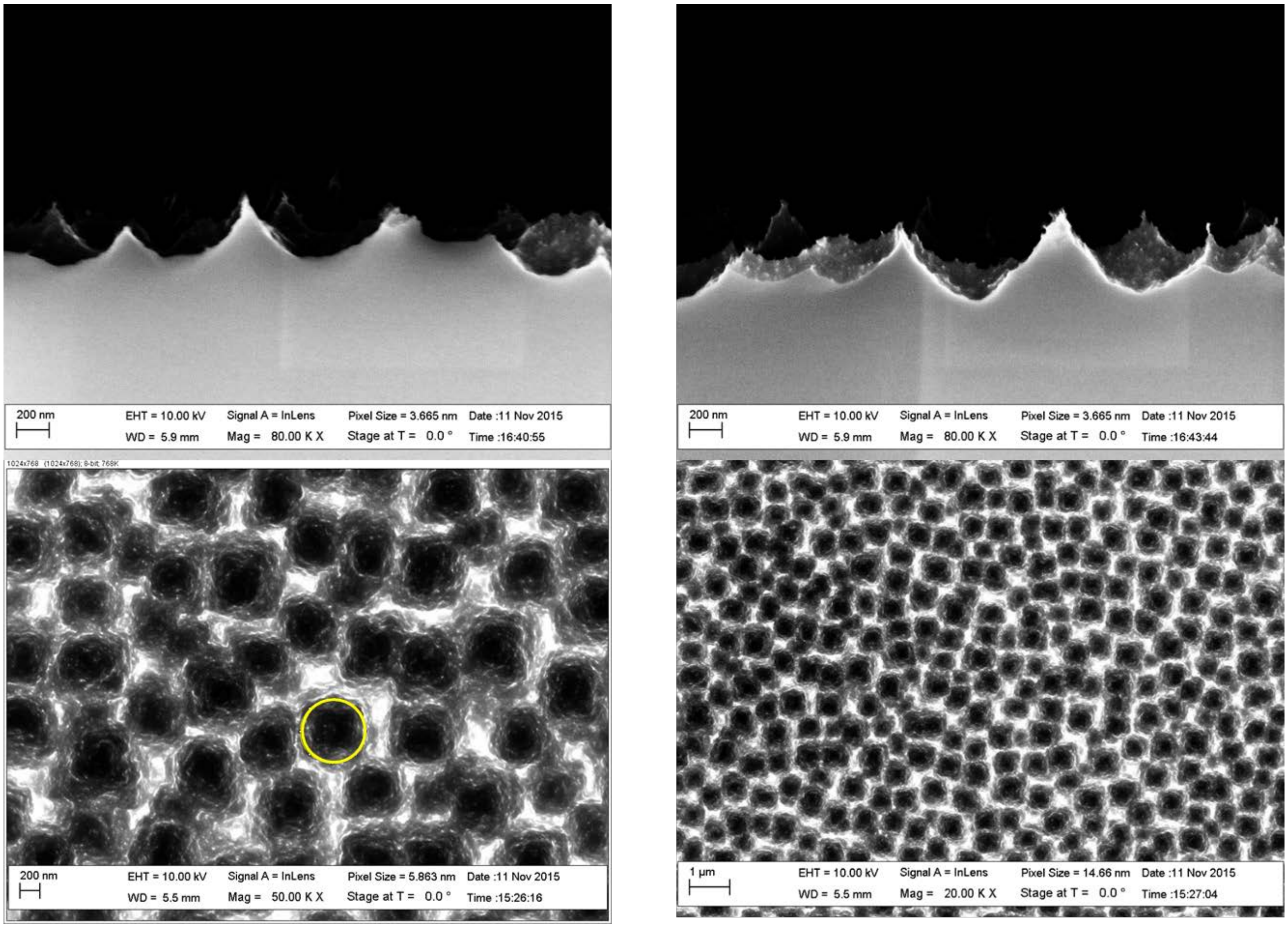


**Figure S1**. SEM images (cross-sectional view and surface view) of uncoated sample 55-10-10 at different resolutions.

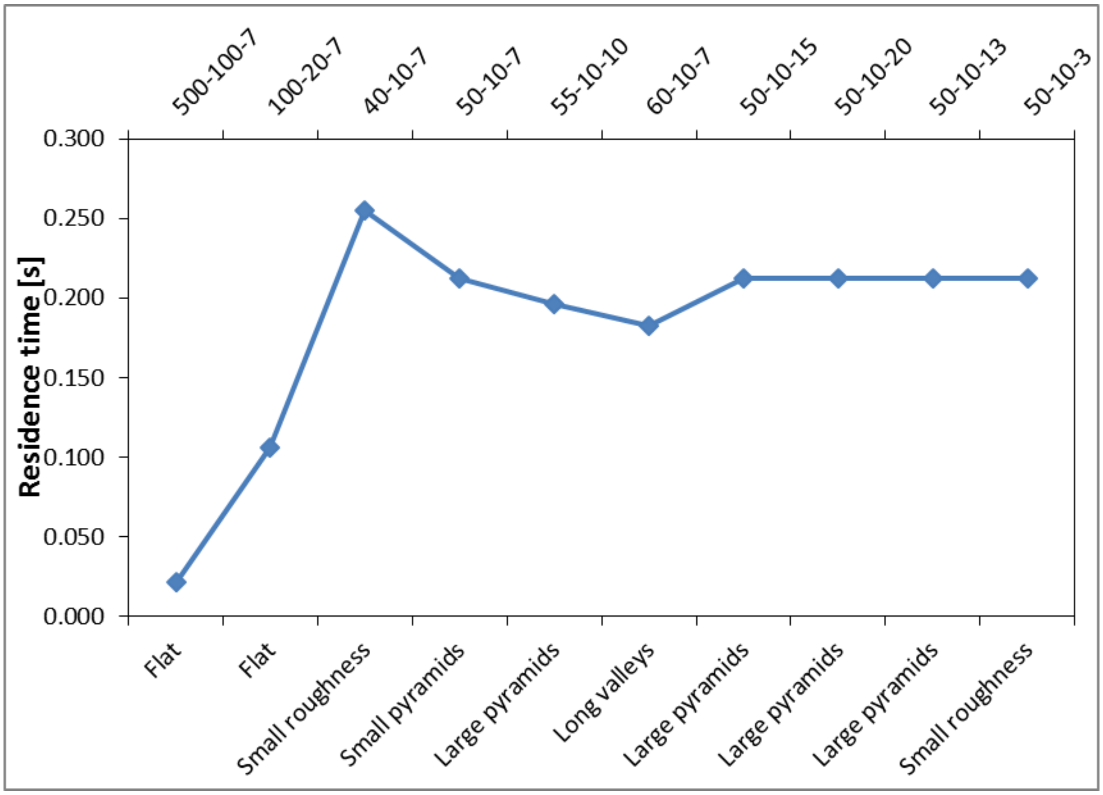


**Figure S2**. Residence time of different etching parameters and resulting structures.

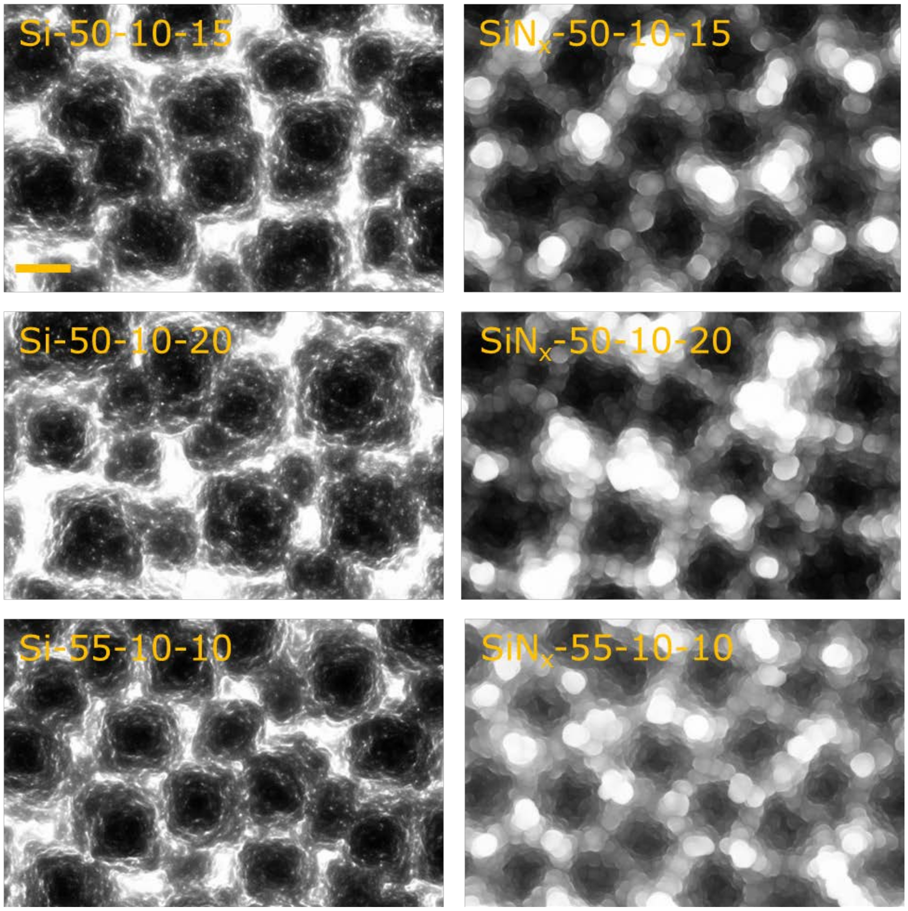


**Figure S3**. SEM images of samples without and with $SiN_x$ coating. The scale bar is 500 nm.

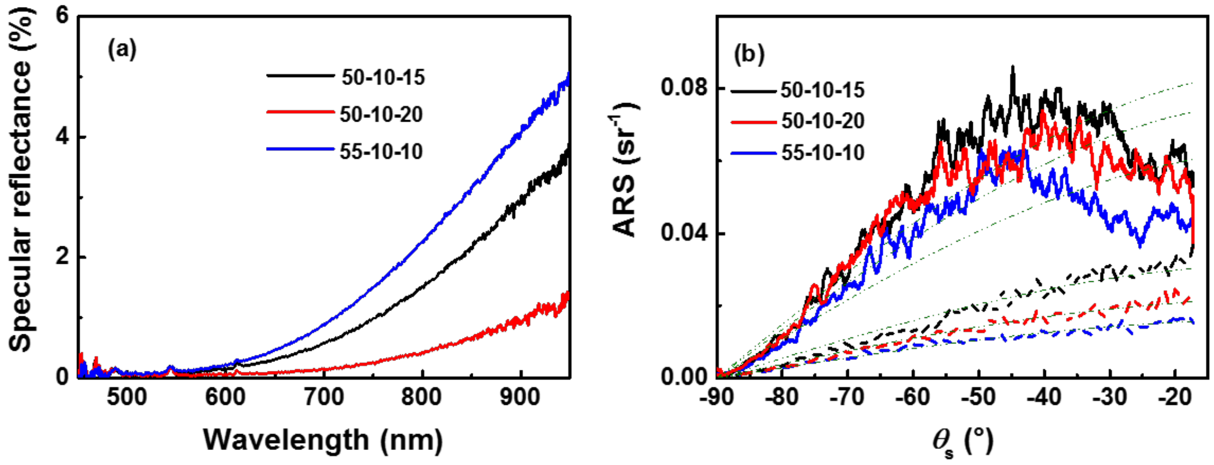

**Figure S4**. (a) Zoomed specular reflectance spectra of inverted pyramids; (b) ARS of inverted pyramids without (solid line) and with (dash line) $SiN_x$ coating. The green dash lines are the corresponding cosine fittings, with the fitting function of $ARS = A \cdot \cos(\theta_s)$.

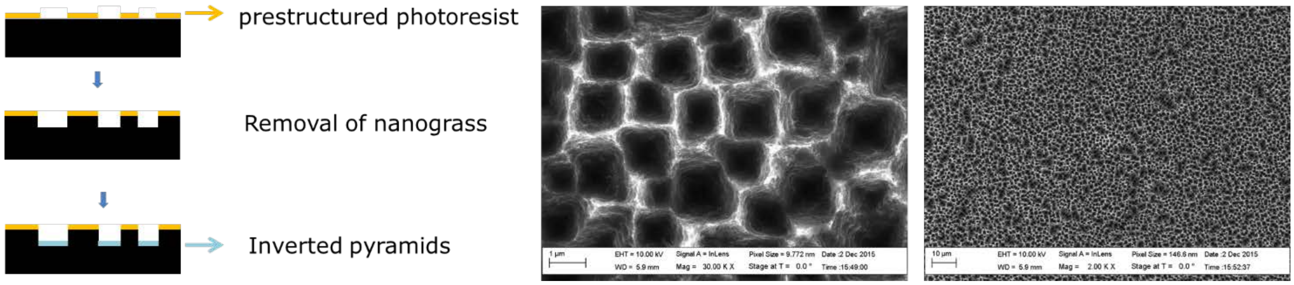


**Figure S5**. (a) Fabrication process flow of the white DTU logo on the black silicon wafer. (b and c) Corresponding SEM images of the logo area of different magnifications.

Fabrication procedure: the black silicon wafer was first coated with a positive photoresist, and exposed through a photomask of the logo shape, and developed. The resulting black silicon wafer with the prestructured photoresist was placed in the RIE chamber, and etched by $SF_6$ and $C_4F_8$ for 12 min to remove the nanograss in the logo area. The total removal is about 2.2 µm. After the removal of the nanograss, the recipe used for sample 55-10-10 was applied to create the inverted pyramids in the logo area.
